# Phytochemical exploration of *Neolitsea pallens* leaves using UPLC-Q-TOF-MS/MS approach

**DOI:** 10.1038/s41598-024-58282-6

**Published:** 2024-04-02

**Authors:** Nisha Thakur, K. Murali, Khushaboo Bhadoriya, Y. C. Tripathi, V. K. Varshney

**Affiliations:** Chemistry and Bio-Prospecting Division, Forest Research Institute, Dehradun, India

**Keywords:** Analytical chemistry, Organic chemistry

## Abstract

*Neolitsea pallens* (D. Don) Momiyama & H. Hara (Family: Lauraceae), commonly known as Pale Litsea, is an evergreen small tree, distributed in India at altitudes of 1500–3000 m. Traditionally utilized for various purposes, its leaves and bark are used as spices, and the plant is valued in preparing a hair tonic from freshly pressed juice. Secondary metabolites of the leaves have not comprehensively been analysed so far. The objective of the study was to determine the chemical composition of the leaves by analysing their 25% aqueous methanol extract with the aid of ultra-performance liquid chromatography quadrupole time of flight tandem mass spectrometry. Overall, 56 compounds were identified in the study. Phenolics represented by phenolic acids, phenolic glycosides, proanthocyanidins, and flavonoids were the main components of the extract.

## Introduction

The *Neolitsea* genus, belonging to the Lauraceae family, comprises woody perennial shrubs and trees found in evergreen forests. It encompasses 85 species distributed throughout tropical and subtropical regions of Southeast Asia. Economically, *Neolitsea* species are rich sources of medicine, timber, spices, and perfumes^1^. Apart from serving as a reservoir of valuable resources, the genus serves its good reputation in folk medicines. *Neolitsea* species plants have been used in traditional medicine for the treatment of various illnesses including furuncle, carbuncle, edema, fractures, eruptions on fingers, and rheumatic arthralgia and especially, the bark and leaves of *N. cassia* were used for the treatment of fractures^1^. Ethanolic extracts of leaves of *N. sericea var. aurata* showed the presence of 14 new alkaloids using the HPLC-SPE-NMR technique^2^ and 37 known alkaloids were reported from this genus^3^. The genus is rich in alkaloids, mainly isoquinoline, and aporphine^4^, sesquiterpenes, triterpenes, and steroids^3^.

*Neolitsea pallens* (D. Don) Momiyama & H. Hara (Family: Lauraceae), commonly known as Pale Litsea, is an evergreen tree. In India, it is mainly distributed in the states of Jammu & Kashmir, Himachal Pradesh, and Uttarakhand, thriving at altitudes ranging from 1500-3000 m^5^. *N. pallens* holds significant ethnobotanical value, being utilized for various purposes*.* The leaves and bark serve as spices, and the freshly pressed juice of the plant is esteemed for its efficacy as a hair tonic^6^. Additionally, the local inhabitants in Himachal Pradesh collect its stems or leaves for fuel in household food preparation^7^. Despite its diverse uses, the chemistry of *N. pallens* remains largely unexplored. Previous investigations focused on the steam-distilled volatile oils composition of its leaves, bark, and fruits using GC and GC-MS^8^. The leaf oil was rich in sesquiterpenoids with furanogermenone (30.6%), β-caryophyllene (19.3%) and germacrene D (12.7%) as major constituents. The bark oil was dominated by oxygenated sesquiterpenoids represented by furanogermenone (59.1%), germacrone (9.3%), 10-epi-γ-eudesmol (7.8%) and curcumenol (5.3%). Furanogermenone (54.8%), trans-β-ocimene (8.8%), sabinene (6.4%) and germacrene D (4.0%) constituted the major proportion of the fruit oil. Furanogermenone constituted the highest proportion in all three oils. However, the non- volatile chemical constituents present in the leaves have remained unknown until now.

Given that leaves are often the primary site for biosynthesis and accumulation of secondary metabolites, including bioactive compounds like phenolic compounds and terpenoids, studying the leaves of *N. pallens* is essential for understanding their chemical composition. Additionally, leaves are renewable and readily accessible plant parts, making them suitable for phytochemical studies aimed at identifying bioactive compounds for pharmaceutical purposes. Phenolics, which are known for their bioactive properties and associated health benefits, have not been previously reported in the *Neolitsea* genus^9^. Therefore, the objective of this study was to comprehensively investigate the chemistry of *N. pallens* leaves, with a focus on phenolic compounds. To achieve this objective, 25% aqueous methanol extract of the leaves was analysed by ultra performance liquid chromatography-quadrupole-time-of-flight tandem mass spectrometry (UPLC-Q-TOF-MS/MS), a state of art technique in plant metabolomics.

## Material and methods

*Chemicals:* Methanol, chloroform, and n-hexane (LR grade) were purchased from Merck (India). Methanol and water (LC–MS grade) were purchased from J.T. Baker (Center Valley, PA, USA) and Carlo Erba (Val de Reuil, France), respectively. Formic acid was obtained from Sigma-Aldrich (Saint-Quentin Fallavier, France) and fresh distilled water was prepared in the laboratory using a distillation unit.

### Collection of plant material and its extraction

**Figure 1:**
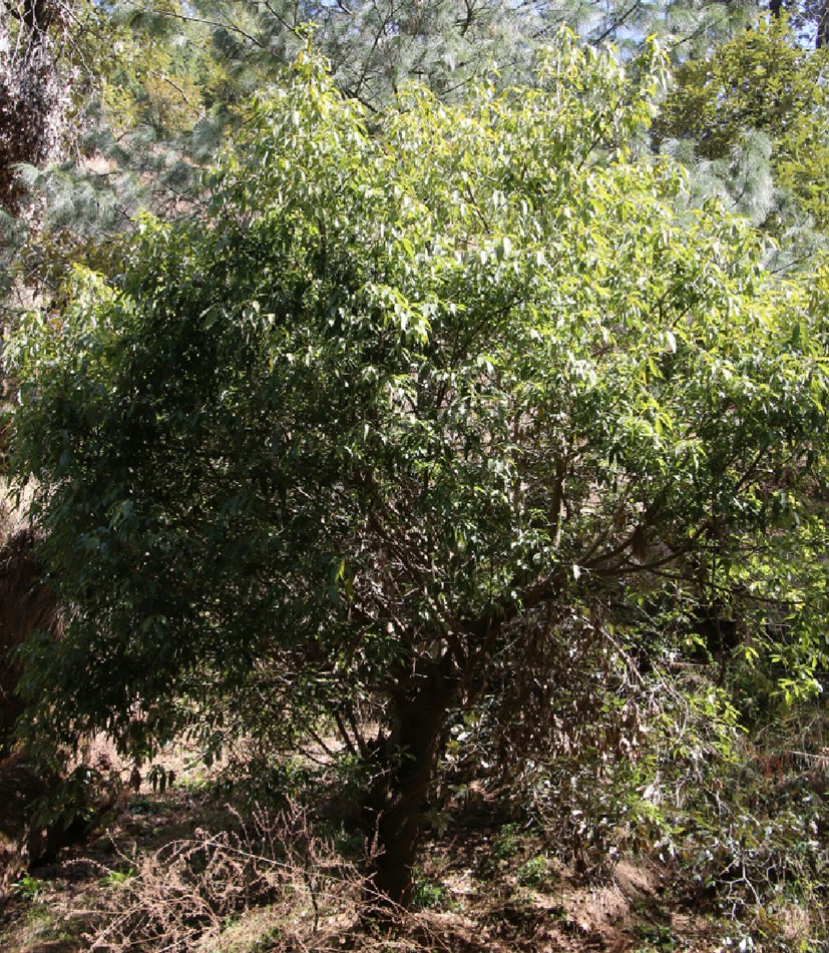
*Neolitsea pallens* growing in Majhrana, Himachal Pradesh, India.

*Plant sample:* The fresh leaves of *N. pallens* (20 accessions) were collected from their natural habitat in Majhrana, Himachal Pradesh, India (Fig. 1), at an altitude of 2316 m (31°12.014″ N and 77°18.288″ E) in the month of March, 2021. The plant collection adhered to all relevant guidelines and the permission was obtained from the Divisional Forest Officer, Shimla, Himachal Pradesh, India. The plant specimen (flowering stage) was authenticated at Systematic Botany Discipline of Forest Botany Division of the ICFRE-Forest Research Institute (FRI) Dehradun. The voucher specimen AN: 172,813 was also deposited in the herbarium of the Systematic Botany Discipline.

*Extraction of the leaves and sample preparation:* The leaves collected from 20 accessions were pooled, and made into a composite sample which was lyophilized at − 40 °C and milled into a powder using a mixer grinder. The leaves were extracted sequentially using n-hexane, chloroform, and 25% aqueous methanol, respectively. Primarily, 5 g of powdered leaves were extracted with 50 mL of n-hexane using ultrasonication for 1 h then the supernatant was filtered through Whatman no.1 filter paper. The residue was re-extracted two more times with fresh solvent. The filtrates were combined and passed through charcoal black for removal of chlorophyll from the extract and concentrated in a vacuum using a rotary evaporator (Buchi, Switzerland) at the temperature 40 °C (279.42 mmHg). The residue was subsequently subjected with chloroform followed by 25% aqueous methanol three times and the liquid extracts, after treatment with charcoal black for removal of chlorophyll, were concentrated in vacuum using a rotary evaporator at the temperatures 42 °C (400 mmHg) and 50 °C (406 mmHg), respectively. All the extracts were collected individually and stored at 2–4 °C for their chemical examinations. The yield (%) of 25% aqueous methanol extract was found to be 15.85 ± 1.26 (Mean ± S.D). A fresh solution (100 ppm) of the extract was prepared in methanol and filtered through a 0.22-μm polyvinylidene difluoride (PVDF) membrane (MILLEX GV filter unit) and transferred into a UPLC autosampler vial prior to LC–MS analysis.

*UPLC-Q-TOF-MS/MS analysis of 25% aqueous methanol extract*: UPLC-QTOF-MS/MS analysis of 25% aqueous methanol extract was conducted using an Agilent 6546 system, a Quadrupole time-of-flight (QTOF) mass spectrometer coupled with an Agilent 1290 Infinity II UPLC system via Dual AJS (ESI) electrospray ionization Source (Agilent Technologies, Santa Clara, CA, 95,051, US). The Agilent 1290 UPLC system consisted of a quaternary pump (G1311A), an online vacuum degasser (G1322A), an auto sampler (G1329A), and a diode array detector (G1315D). Chromatographic separation of the extract was achieved using an Agilent ZORBAX RRHD Eclipse Plus reversed phase C_18_ column (2.1 × 100 mm, 1.8 μm). The mobile phase consisted of 0.1% formic acid in water (solvent A) and 100% methanol (solvent B), with a flow rate of 0.4 mL/min under the following gradient elution program: 5% B at 0–2 min, 5–20% B at 2–6 min, 20–45% B at 6–18 min, 45–95% B at 18–25 min, and a post time of 3 min, resulting in a total run time of 28 min. The sample injection volume was 5 μL, and the column temperature was set at 35 °C. Mass spectrometric analysis was carried out on an Agilent 6520 QTOF mass spectrometer in Negative ESI mode. The resolving power of the QTOF analyzer was set above 10,000 (FWHM, full width at half maximum), and spectra were acquired within a mass range of m/z 100–1700. Nitrogen gas was used for nebulizing, drying in the ionization source, and also for collision in the CID (Collision-induced Dissociation) cell. The sheath gas temperature was maintained at 350 °C, with a flow rate of 11 L/min, and the fragmentor voltage was set to 100 V. The capillary voltage was adjusted to 3500 V, the nebulizer pressure to 35 psi, and the drying gas flow rate to 10 L/min. Collision energy values for MS/MS experiments were fixed at 10 eV, 20 eV, and 40 eV for all the selected masses. The total number of injections was 7, consisting of 3 blank injections followed by 3 sample injections for MS analysis, and then 1 sample injection for Auto MS/MS analysis. Data analysis was performed using Agilent Mass Hunter Workstation Data Acquisition software version B.03.01 (version 10.0 Qualitative Analysis, Agilent Technologies, Santa Clara, CA, 95051, USA), which generated the molecular formula with a mass accuracy limit of 5 ppm (related to the contribution to mass accuracy, isotope abundance, and isotope). To obtain chemical structure information, the following databases were consulted: PubChem (https://pubchem.ncbi.nlm.nih.gov), ChemSpider (http://www.chemspider.com/), Food Database (http://foodb.ca/), Mass Bank (http://www.massbank.jp/), and Human Metabolome Database (https://hmdb.ca/).

## Results

**Figure 2:**
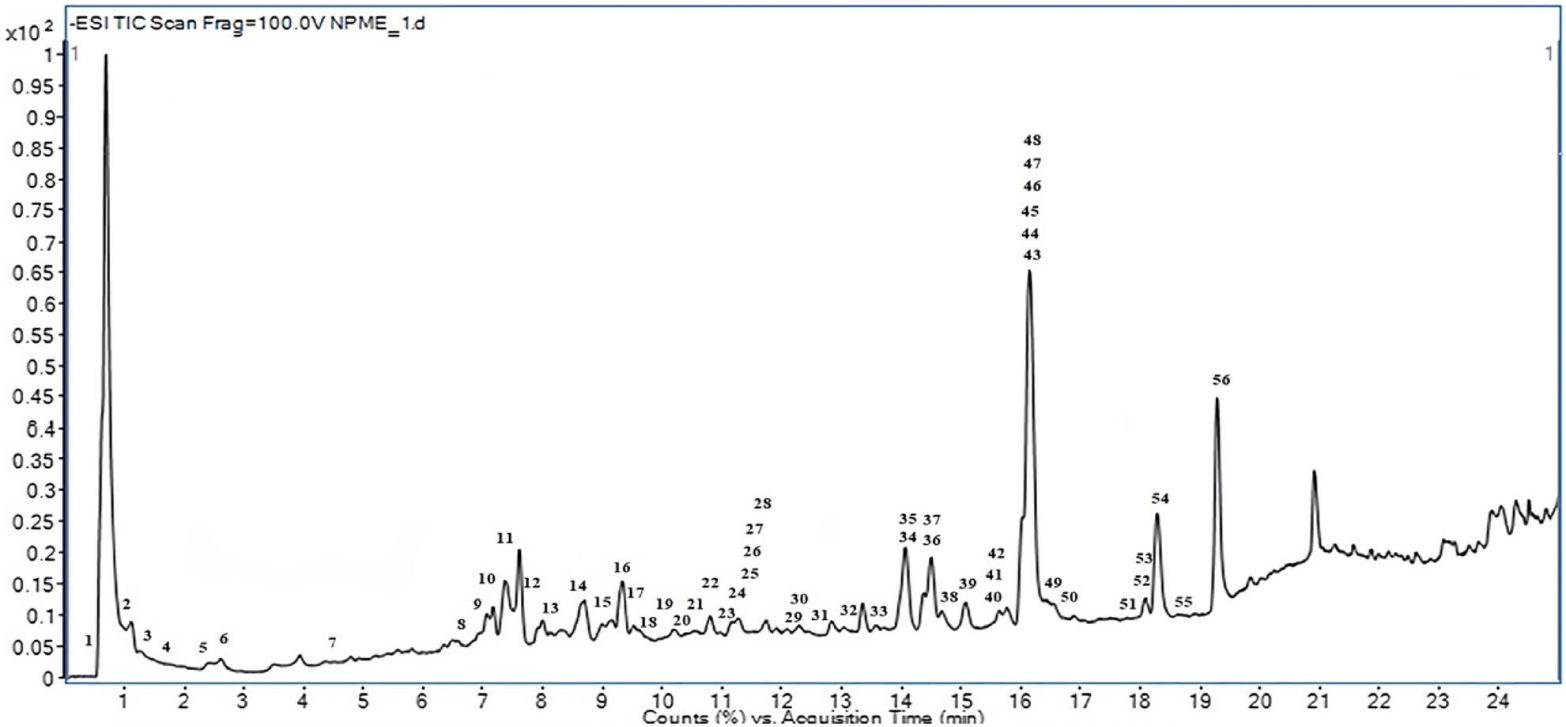
Total ion chromatogram of 25% aqueous methanol extract of *Neolitsea pallens* leaves using negative ionization mode in UPLC-QTOF-MS/MS technique.

In the present investigation, the metabolite profiling of the 25% aqueous methanolic extract of *N. pallens* leaves was elucidated by employing reverse phase UPLC-QTOF-MS and tandem mass spectrometry (MS/MS) under negative ionization mode. Figure 2 depicts the total ion chromatogram of the chemical compounds and the assigned peak numbers correspond to the elution order of the compounds as enumerated in Table 1. A total of 56 chemical compounds were tentatively identified by examination of their MS/MS fragmentation patterns and comparison with the chemical structures of previously reported compounds. Comprehensive MS data for the identified compounds are succinctly presented in Table 1, encompassing retention time (rt), compound class, experimental *m/z*, experimental mass, theoretical mass, mass error in parts per million (ppm), molecular formula, and MS/MS fragment ions by considering the registered mass spectra fragmentation patterns in databases, as well as the observed fragmentation patterns in the current study and previously reported data in the literature. The identified compounds were systematically categorized into distinct classes, comprising four organic acids, five phenolic acids and derivatives, six tannins, two phenylpropanoid glycosides, four lignan glycosides, thirty-one flavonoids, and four miscellaneous compounds. The comprehensive characterization of all 56 compounds in *N. pallens* leaves was conducted for the first time through the analysis of mass spectrometry fragmentation patterns of the compounds utilizing UPLC-Q-TOF–MS/MS technique.

**Table 1:** Retention time and mass spectroscopic characteristic data of constituents identified in 25% aqueous methanol extract of *Neolitsea pallens* leaves using negative ionization mode in UPLC-QTOF-MS/MS technique.

Compound no	Retention time (Min.)	[M-H]^−^ *m/z*	Experimental mass	Theoretical mass	Mass error (ppm)	Molecular formula	Fragment ions	Identified compounds	Class of compounds
1	0.755	191.0197	192.0270	192.0197	0	C_6_H_8_O_7_	173 155 111* 85	Citric acid	Organic acid
2	0.833	133.0141	134.0213	134.0215	− 1.49	C_4_H_6_O_5_	115* 71	Malic acid	Organic acid
3	1.240	203.0192	204.0265	204.0270	-2.4506	C_7_H_8_O_7_	184 140 97*	Daucic acid	Organic acid
4	1.371	117.0191	118.0264	118.0266	-1.6945	C_4_H_6_O_4_	99* 73	Succinic acid	Organic acid
5	2.450	191.0558	192.0630	192.0631	-1.5619	C_7_H_12_O_6_	173 129* 111	Quinic acid	Phenolic acid
6	2.633	331.0664	332.0737	332.0743	− 1.8068	C_13_H_16_O_10_	168* 169 125 124	3-Glucogallic acid	Tannin
7	4.464	331.1028	332.1099	332.1107	− 2.4088	C_14_H_20_O_9_	168 153*	Leonuriside A	Phenolic glycoside
8	6.893	457.1344	458.1417	458.1424	− 1.5279	C_20_H_26_O_12_	163* 119	cis-*p*-coumaric acid 4-[apiosyl-(1- > 2)-hexoside]	Phenolic acid derivative
9	7.158	353.0872	354.0945	354.0950	− 1.4120	C_16_H_18_O_9_	191* 163	Chlorogenic acid	Phenolic acid
10	7.220	577.1345	578.1418	578.1424	− 1.0378	C_30_H_26_O_12_	425 407 289* 125	Procyanidin B5 or Procyanidin B8	Condensed Tannin
11	7.364	863.1828	864.1893	864.1901	− 0.9257	C_45_H_36_O_18_	711 411 289*	Procyanidin type 1A1B (Isomer-I)	Condensed Tannin
12	7.966	385.1133	386.1207	386.1212	− 1.2949	C_17_H_22_O_10_	223* 179	1-*O*-Sinapoyl glucose (Isomer-1)	Phenolic acid derivative
13	8.571	401.1456	402.1522	402.1525	− 0.7459	C_18_H_26_O_10_	269*	Benzyl *β*-primeveroside	Benzyl glycoside derivative
14	8.625	519.1712	520.1784	520.1792	− 1.5379	C_22_H_32_O_14_	-	Citrusin F	Phenylpropanoid glycoside
15	9.324	441.1758	442.1831	442.1838	− 1.5830	C_21_H_30_O_10_	133	Lusitanicoside	Phenylpropanoid glycoside
16	9.426	385.1134	386.1208	386.1212	− 1.0359	C_17_H_22_O_10_	325 223 179	1-*O*-Sinapoyl glucose (Isomer -II)	Phenolic acid derivative
17	9.648	521.2019	522.2091	522.2101	− 1.9149	C_26_H_34_O_11_	359* 344 313	Isolariciresinol-*O*-glucoside (Isomer-I)	Lignan glycoside
18	9.751	521.2020	522.2094	522.2101	− 1.3404	C_26_H_34_O_11_	359 329*	Isolariciresinol*O*-glucoside (Isomer-II)	Lignan glycoside
19	10.133	609.1476	610.1548	610.1533	2.4584	C_27_H_30_O_16_	300* 271 255 151	Quercetin-*O*- rhamnoside -hexoside (I)/ Rutin-I	Flavonol
20	10.199	609.1454	610.1527	610.1533	− 0.9833	C_27_H_30_O_16_	300* 255 271 178 151	Quercetin-*O*- rhamnoside -hexoside (II)/ Rutin-II	Flavonol
21	10.898	581.2231	582.2305	582.2312	− 1.2022	C_28_H_38_O_13_	419* 404	Lyoniresinol 9-*O*-glucoside	Lignan glycoside
22	10.91	331.0456	332.0528	332.0532	− 1.2046	C_16_H_12_O_8_	151* 179	Laricitrin	Flavonol
23	11.258	575.1193	576.1262	576.1265	− 0.5207	C_30_H_24_O_12_	449 423 285* 289 125	Procyanidin A2	Proanthocyanidin
24	11.398	863.1822	864.1894	864.1901	− 0.8100	C_45_H_36_O_18_	711 575* 451 287 125	Procyanidin type 1A1B (Isomer-II)	Condensed Tannin
25	11.723	593.1511	594.1580	594.1584	− 0.6732	C_27_H_30_O_15_	447 327 285*	Kaempferol-*O*-rhamnosyl-*O*-hexoside, isomer-I/ Astragalin 7-rhamnoside	Flavonol
26	11.865	593.1505	594.1578	594.1584	− 1.0098	C_27_H_30_ O_15_	285* 255 161	Kaempferol-*O*-rhamnosyl-*O*-hexoside, isomer-II/ Astragalin 7-rhamnoside	Flavonol
27	11.881	523.2177	524.2252	524.2257	− 0.9537	C_26_H_36_O_11_	361* 179 146	Mascaroside	Naphthofuran
28	11.950	577.1344	578.1417	578.1424	− 1.2107	C_30_H_26_O_12_	451 425 407 289 125	Procyanidin B8 or Procyanidin B5	Condensed tannin
29	12.789	521.2020	522.2094	522.2101	− 1.3404	C_26_H_34_O_11_	359* 329 341	Isolariciresinol-*O*-glucoside (Isomer-III)	Stilbene glycoside
30	12.967	595.1296	596.1371	596.1377	− 1.0064	C_26_H_28_O_16_	300* 271	Quercetin-3-*O*-arabinohexoside (Isomer-I)	Flavonol
31	13.161	595.1297	596.1369	596.1377	− 1.3419	C_26_H_28_O_16_	300*	Quercetin-3-*O*-arabinohexoside (Isomer-II)	Flavonol
32	13.820	609.1451	610.1527	610.1533	− 0.9833	C_27_H_30_O_16_	300* 301 463 299	Quercetin-*O*- rhamnoside -hexoside (III)/ Rutin-III	Flavonol
33	14.067	463.0879	464.0951	464.0954	− 0.6464	C_21_H_20_O_12_	300* 271 151	Quercetin-*O*-hexoside, isomer-I	Flavonol
34	14.188	593.1503	594.1576	594.1584	− 1.3464	C_27_H_30_O_15_	447 284* 255 163	Kaempferol-*O*-rhamnosyl-hexoside, Isomer-I	Flavonol
35	14.354	463.0875	464.0947	464.0954	− 1.5083	C_21_H_20_O_12_	300* 271 179 151	Quercetin-*O*-hexoside , isomer-II	Flavonol
36	14.479	609.1456	610.1528	610.1533	− 0.8194	C_27_H_30_O_16_	300* 271 151	Quercetin-*O*- rhamnoside -hexoside (IV)/ Rutin-IV	Flavonol
37	14.723	433.0771	434.0843	434.0849	− 1.3822	C_20_H_18_O_11_	300* 271	Guaijaverin (Isomer-I)	Flavonol
38	15.100	433.0770	434.0843	434.0849	− 1.3822	C_20_H_18_O_11_	300* 271 178 151	Guaijaverin (Isomer-II)	Flavonol
39	15.639	447.0928	448.1001	448.1005	− 0.8926	C_21_H_20_O_11_	447 284* 255 227	Kaempferol -*O*-hexoside (Isomer-I)	Flavonol
40	15.717	593.1501	594.1576	594.1584	− 1.3464	C_27_H_30_O_15_	447 285*	Kaempferol-*O*-rhamnosyl-hexoside, Isomer-II	Flavonol
41	15.938	447.0926	448.0998	448.1005	− 1.5621	C_21_H_20_O_11_	301* 300 271 243	Quercetin-*O*-rhamnoside (Isomer-I)	Flavonol
42	16.120	579.1353	580.1425	580.1428	− 0.5171	C_26_H_28_O_15_	300 271	Quercetin-*O* -rhamnosyl-arabinopyranoside (Isomer-I)	Flavonol
43	16.194	447.0930	448.1003	448.1005	− 0.4463	C_21_H_20_O_11_	301* 271 243	Quercetin-*O*-rhamnoside (Isomer-II)	Flavonol
44	16.255	447.0934	448.1006	448.1005	0.2232	C_21_H_20_O_11_	285 284* 255 227	Kaempferol-*O*-hexoside (Isomer-II)	Flavonol
45	16.416	593.1502	594.1575	594.1584	− 1.5147	C_27_H_30_O_15_	447 285* 255	Kaempferol-*O*-rhamnosyl-hexoside, Isomer-III	Flavonol
46	16.463	417.0820	418.0895	418.0899	− 0.9567	C_20_H_18_O_10_	284* 255 227	Kaempferol -*O*-arabinoside (Isomer-I)	Flavonol
47	16.581	579.1348	580.1421	580.1428	− 1.2066	C_26_H_28_O_15_	447 300	Quercetin-*O*-rhamnosyl-arabinopyranoside (Isomer-II)	Flavonol
48	16.606	593.1504	594.1577	594.1584	− 1.1781	C_27_H_30_O_15_	447 285* 255 151	Kaempferol-*O*-rhamnosyl-hexoside, Isomer-IV	Flavonol
49	16.728	417.0820	418.0892	418.0899	− 1.6742	C_20_H_18_O_10_	284* 255 227	Kaempferol-*O*-arabinoside (Isomer-II)	Flavonol
50	17.977	563.1399	564.1472	564.1479	− 1.2408	C_26_H_28_O_14_	284* 255	Kaempferol-*O*-rhamnoside- *O*-xyloside	Flavonol
51	18.287	431.0978	432.1050	432.1056	− 1.3855	C_21_H_20_O_10_	285* 255 227	Kaempferol-*O*-rhamnoside, Isomer-I/Afzelin-I	Flavonol
52	18.586	301.0350	302.0422	302.0422	0	C_15_H_10_O_7_	151* 107	Quercetin	Flavonol
53	18.644	431.0982	432.1054	432.1056	− 0.4628	C_21_H_20_O_10_	285* 255 227	Kaempferol-*O*-rhamnoside, Isomer-II/Afzelin-I	Flavonol
54	20.121	593.1290	594.1361	594.1373	− 2.0197	C_30_H_26_O_13_	285* 284* 165 121	Tiliroside	Flavonol
55	22.489	723.1711	724.1783	724.1792	− 1.2427	C_39_H_32_O_14_	285* 559 163 119	2'',3''-Di-*O*-*p*-coumaroylafzelin/ Platanoside	Flavonol
56	24.87	297.2433	298.2505	298.2507	− 0.6705	C_18_H_34_O_3_	183*	Ricinoleic acid	Fatty acid

*Base peak or characteristic peak.

## Discussion

*Organic acids*. Four compounds were tentatively identified as organic acids. Compound 1 was detected at a retention time (rt) of 0.755 min, appearing as an [M-H]^−^ ion at *m/z* 191.0197. The molecular formula was assigned as C_6_H_8_O_7_ using Electrospray Ionization High-Resolution Mass Spectrometry (ESI-HRMS). Tandem mass spectral analysis revealed fragment ions at *m/z* 173 [M-H-H_2_O]^−^, 155 [M-H-2H_2_O]^−^, 111 [M-H-2H_2_O-CO_2_]^−^, and 85 [M-H-2H_2_O-CO_2_-C_2_H_2_]^−^. Integrating the mass spectrometry data and interpreting the MS/MS fragmentation pattern (Scheme 1 [supporting information]), this compound was identified as citric acid^10^.

Compound 2, eluting at a retention time of 0.833 min, was detected as an [M-H]^−^ ion at *m/z* 133.014. The ascribed molecular formula, C_4_H_6_O_5_ was established through precise mass determination. Upon fragmentation, it yielded product ions at *m/z* 115 [M-H-18Da]^−^ and *m/z* 71 [M-H-18-44Da]^−^, indicative of the sequential loss of a water molecule (−H_2_O) followed by the elimination of a carbon dioxide molecule (−CO_2_) from the precursor ion. Accordingly, through comprehensive analysis of the acquired MS/MS data and cross-referencing with published literature, this compound was identified as malic acid^11^. The elucidated fragmentation pattern of this compound is depicted in scheme 2 (supporting information).

Compound 3 (rt 1.240 min) was detected as an [M-H]^−^ ion at *m/z* 203.0192 with a molecular formula C_7_H_8_O_7_ and it was tentatively identified as daucic acid based on its fragmentation pattern (Scheme 3 [supporting information]) and online database (PubChem and FooDB). The MS/MS spectra exhibited distinctive product ions at *m/z* 184 [M-H-18Da]^−^, *m/z* 140 [M-H-18-44Da]^−^, and *m/z* 97 [M-H-18-44-44Da]^−^, attributed to the sequential loss of H_2_O, H_2_O + CO_2_, and H_2_O + 2CO_2_ from the parent ion. Notably, the fragment ion at *m/z* 97 [M-H-H_2_O-2CO_2_]^−^ emerged as the base peak in the spectrum.

Compound 4 (rt 1.371 min) displayed a [M-H]^−^ molecular ion at *m/z* 117.0191and tentatively identified as succinic acid (C_4_H_6_O_4_) through MS spectral comparison with published data^12^. The MS/MS spectra revealed distinct fragment ions at *m/z* 99 [M-H-18Da]^−^ and *m/z* 73 [M-H-44Da]^−^, indicative of sequential losses of H_2_O and CO_2_, respectively (Scheme-4 [supporting information]).

*Phenolic acids and derivatives.* Five compounds were identified within this class, comprising two phenolic acids and three derivatives of phenolic acids. Compound 5 (rt 2.450 min) displayed a [M-H]^−^ precursor ion at *m/z* 191.0191, with a molecular formula C_7_H_12_O_6_. The MS/MS fragment ions at *m/z* 173, *m/z* 129, and *m/z* 111 corresponded to neutral losses of H_2_O, H_2_O + CO_2_, and 2H_2_O + CO_2_, respectively. The current MS/MS data, along with reported literature^13–15^, led to the identification of compound 5 as quinic acid. The proposed fragmentation pattern is illustrated in scheme 5 (supporting information). Compound 8 (rt 6.893 min) displayed an [M-H]^−^ ion at *m/z* 457.1344, with the molecular formula C_20_H_26_O_12_ determined by ESI-HRMS. The MS/MS spectra revealed product ions at *m/z* 163 and 119. A characteristic fragment ion at *m/z* 163 [M-H-C_11_H_18_O_9_]^−^, representing the loss of deoxyribosyl-rhamnosyl (−294 Da) moiety, further fragmented into *m/z* 119 by the loss of a CO_2_ molecule (Scheme 8 [supporting information]). Considering the current MS/MS data and previously reported literature, this compound was identified as cis-*p*-coumaric acid 4-[apiosyl-(1- > 2)-hexoside]^16–19^. Compound 9 (rt 7.158 min) with the molecular formula C_16_H_18_O_9_, exhibited a precursor ion [M-H]^−^ at *m/z* 353.0872. Upon fragmentation, it yielded a distinct and intense fragment ion at *m/z* 191 [M-H-C_9_H_6_O_3_]^−^ resulting from the neutral loss of the *p*-coumaric acid moiety (−162 Da). This fragment was identified as quinic acid [Scheme 9 [supporting information]. Consequently, the compound (9) was identified as chlorogenic acid, supported by the comprehensive analysis of MS/MS data and corroborated by findings from previous reports^14,20,21^. Compounds 12 (rt: 7.966 min) and 16 (rt: 9.426 min) were detected as [M-H]^−^ ions at *m/z* 385.1133, and their molecular formula was determined to be C_17_H_22_O_10_ through HRMS analysis. Both compounds were recognized as isomers, featuring similar fragment ions at *m/z* 223, 179, 208, 164, and 149, corresponding to common neutral losses. Notably, the MS/MS analysis revealed fragment ions at *m/z* 223 [M-H-163Da]^−^, attributed to the loss of the sugar moiety. Subsequent fragmentation produced daughter ions at *m/z* 208, 179, 164, and 149 through the loss of neutral molecules CH_3_, CO_2_, CO_2_ + CH_3_, and CO_2_ + 2CH_3_, respectively (Scheme 12 and Scheme 16 [supporting information]). The collective evidence from MS/MS data and previously reported literature^22^, allowed the confident identification of compounds 12 and 16 as isomers of 1-*O*-sinapoyl glucose.

*Tannins*. Six compounds were identified in this class, including one hydrolyzable tannin and five condensed tannins, commonly referred to as proanthocyanidins. Compound 6, eluted at a retention time of 2.633 min, exhibited a molecular ion [M-H]^−^ at *m/z* 331.0664, corresponding to the molecular formula C_13_H_16_O_10_. The MS/MS spectra unveiled distinct product ions at *m/z* 168 [M-H-164Da]^−^ and *m/z* 125 [M-H-C_6_H_12_O_5_-44Da]^−^. The characteristic fragment ion was observed at *m/z* 168 due to loss of rhamnose [-C_6_H_12_O_5_] and it was identified as gallic acid (Scheme 6 [supporting information]). Consequently, based on the acquired MS/MS data and corroborating literature^23,24^, Compound 6 was identified as a 3-glucogallic acid. Compounds 10 (rt: 7.220 min) and 28 (rt: 11.950 min) were detected as [M-H]^−^ ions at *m/z* 577.1345, featuring the molecular formula C_30_H_26_O_12_. They were tentatively identified as proanthocyanidin dimers based on previously reported literature^25–28^. The MS/MS spectra of both compounds exhibited similar fragment ions at *m/z* 425, 289, 451, 299, 125, and 407, generated through a specific fragmentation reaction outlined in Scheme 10 (supporting information). The product ion at *m/z* 425 [M-H-152Da]^−^ was produced by retro-Diels–Alder (RDA) fission, further fragmented into 407 [M-H-152-18Da]^−^ due to the neutral loss of an H_2_O molecule. Additionally, the fragment ion at *m/z* 289 [M-H-288Da]^−^ resulted from quinone methide (QM) fission, while ions at *m/z* 125 [M-H-126Da]^−^ and 451 [M-H-126Da]^−^ were formed by heterocyclic ring fission from the parent ion. According to published reports^25–28^, the current MS/MS data, and a significant difference in retention time, compounds 10 and 28 were tentatively identified as isomers of procyanidin B5 and procyanidin B8, respectively (Scheme 10 [supporting information]).

Compounds 11 (rt: 7.364 min) and 24 (rt: 11.398 min) were detected with a precursor [M-H]^−^ ion at *m/z* 863.182, and they were assigned the molecular formula C_45_H_36_O_18_ based on accurate mass measurements. Both compounds were identified as isomers of proanthocyanidin trimers, exhibiting similar product ions at *m/z* 711 [M-H-152Da]^−^, indicative of a loss of 152 Da through RDA fission. In the MS/MS spectra of compound 24, product ion at *m/z* 737 was observed via heterocyclic ring fission, and ions at *m/z* 575 and 287 resulted from QM fission, respectively. Compound 11 exhibited product ion at *m/z* 411, possibly through QM fission followed by RDA fission. The proposed fragmentation pathway is illustrated in Schemes 11 and 24 (supporting information). Based on the current MS/MS data and information from previously reported literature^25,29–35^, these two compounds were tentatively identified as isomers of procyanidin type 1A1B.

Compound 23 (rt: 11.258 min) was detected as an [M-H]^−^ ion at *m/z* 575.1193, and its molecular formula was assigned as C_30_H_24_O_12_ based on accurate mass measurements. This compound was identified as a proanthocyanidin dimer, relying on previously reported literature^25^. The tandem mass spectra produced fragment ions at *m/z* 423, 449, 285, 289, and 125. These fragment ions were produced through specific fragmentation pathways (Scheme 22 [supporting information]). The fragment ion at *m/z* 423 [M-H-152Da]^−^ was formed through RDA fission and *m/z* 285 was formed due to QM fission. Additionally, the product ions at *m/z* 449 [M-H-126Da]^−^ and 125 [M-H-150Da]^−^ were produced through heterocyclic ring fusion. Based on the current MS/MS data and information from previously reported literature, this compound could be identified as proanthocyanidin A2.

*Lignan glycoside.* Three compounds, 17 (rt: 9.648 min), 18 (rt: 9.751 min), and 29 (rt: 12.789 min), were detected as [M-H]^−^ ions at *m/z* 521.202, with a molecular formula of C_26_H_34_O_11_. These compounds, were identified as isomers with a common characteristic peak at *m/z* 359 [M-H-162Da]^−^, resulting from the loss of a sugar moiety (-C_6_H_10_O_5_). The fragment ion peak at *m/z* 329 [M-H-162-31Da]^−^ was commonly observed in compounds 18 and 29 due to the loss of a methoxy group (–OCH_3_) from *m/z* 359. Additionally, the daughter ion at *m/z* 341 [M-H-162-18Da]^−^ was observed in compound 29 due to the loss of a neutral water molecule (-H_2_O) from *m/z* 359. In contrast, fragment ions at *m/z* 344 [M-H-162–15Da]^−^ and 313 [M-H-162-15-31Da]^−^ were observed in compound 17, indicating the loss of a methyl group (–CH_3_) followed by a methoxy group (–CH_3_O) from *m/z* 359. The fragmentation patterns of these three compounds were presented in Schemes 17, 18, and 29 (supporting information). According to published reports^36^ and the current MS/MS data, these three compounds were identified as isomers of isolariciresinol-*O*-glucoside, respectively.

Compound 21 (rt: 10.898 min) was observed as an [M-H]^−^ ion at *m/z* 581.2231 with molecular formula (C_28_H_38_O_13_). In the MS/MS spectra, a prominent characteristic fragment ion at *m/z* 419 [M-H-162Da]^−^ was observed, resulting from the loss of the rhamnose moiety (−C_6_H_10_O_5_). Upon further fragmentation, the ensuing fragment ions were observed at *m/z* 404 [M-H-162–15Da]^−^ and *m/z* 373 [M-H-162–15-31Da]^−^, indicative of sequential losses of a methyl group in the radical form (−CH_3_˙) followed by a methoxy group (-CH_3_O˙), respectively (Scheme 20 in the Supporting Information). Considering the current MS/MS data and relevant literature^37^, compound 21 was tentatively identified as lyoniresinol 9-glucoside.

**Figure 3:**
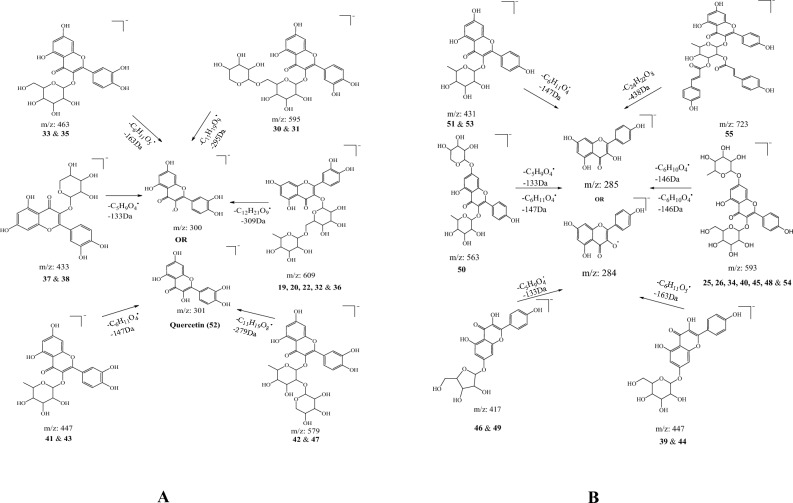
(**A**): Proposed Fragmentation Pathways for Quercetin-Based Derivatives. (**B**): Proposed Fragmentation Pathways for Kaempferol-Based Derivatives.

*Flavonoids.* Flavonoids constitute a diverse and prevalent class of polyphenolic compounds. Chemically, they consist of a 15-carbon skeleton comprising two aromatic rings (A and B) linked by a three-carbon bridge (C). This class represents a paramount group of phenolic compounds, notably abundant in 25% aqueous methanol extract of *N. pallens* leaves. Within this classification, a total of 31 compounds were identified and characterized within the sub-class of flavonols. Particularly, compounds 19, 20, 22, 32, 36, 30, 31, 33, 35, 37, 38, 41, 43, 42, 47, and 52 were identified as quercetin-based derivatives (Fig. 3A), while compounds 25, 26, 34, 40, 45, 48, 54, 39, 44, 46, 49, 50, 51, 53, and 55 were determined to be kaempferol-based derivatives (Fig. 3B).

Four compounds, 19 (rt 10.133 min), 20 (10.199 min), 32 (13.82 min), and 36 (14.479 min), were identified as [M-H]^−^ ions at *m/z* 609.14, sharing the identical molecular formula C_27_H_30_O_16_. These compounds were characterized as isomers, featuring a common characteristic product ion at *m/z* 300 [M-H-309Da]^−^, corresponding to quercetin [M-H]^−^ unit, potentially arising from the loss of a sugar moiety such as glucosyl-rhamnosyl (-C_12_H_21_O_9_). Moreover, in compound 20, an additional product ion at *m/z* 462 [M-H-147Da]^−^ was observed, indicative of the loss of rhamnosyl moiety (-C_6_H_11_O_4_). Through the integration of the present MS/MS data and insights from documented literature^38^, these compounds were identified as isomers of rutin (quercetin-*O*-rhamnoside-hexoside). The fragmentation patterns of these compounds were presented in Scheme 36 [supporting information].

Compound 22 (rt 10.91) exhibited an [M-H]^−^ ion with a *m/z* of 331.0456, and its molecular formula was accurately determined as C_16_H_12_O_8_. The MS/MS spectra revealed fragment ions at *m/z* 151 [M-H-180Da]^−^ and *m/z* 179 [M-H-152Da]^−^, indicative of retro Diels–Alder (RDA) fission. Based on the current MS/MS data and corroborating literature^39^, Compound 22 was tentatively identified as laricitrin, and its proposed fragmentation pattern is depicted in Scheme 21 [supporting information].

Compound 25 (rt 11.723 min), 26 (11.865 min), 34 (14.188 min), 40 (15.717 min), 45 (16.416 min), and 48 (16.606 min) were detected as [M-H]^−^ ions at *m/z* 593.15, all sharing the identical molecular formula C_27_H_30_O_15_, yet eluting at distinct retention times. These compounds were recognized as isomers exhibiting a common characteristic product ion at *m/z* 285 [M-H-162-146Da]^−^, indicative of kaempferol aglycone, achieved through the neutral loss of sugar moieties like hexosyl (162) and rhamnosyl (146). Furthermore, in the MS/MS spectra of compounds 25 and 26, product ions at *m/z* 447 [M-H-146Da]^−^ and 431 [M-H-162Da]^−^ were noted, corresponding to the loss of rhamnosyl and hexosyl, respectively. Based on previously published literature^40^ and the current MS/MS data, compounds 25 and 26 were tentatively identified as isomers of astragalin 7-rhamnoside (kaempferol-*O*-rhamnosyl-*O*-hexoside), while compounds 34, 40, 45, and 48 were discerned as isomers of kaempferol-*O*-rhamnosyl-hexoside. The fragmentation patterns of these compounds are presented in schemes 26 and 23 [supporting information].

Compound 54 (rt 20.121 min) exhibited an [M-H]^−^ ion at *m/z* 593.1290, with its molecular formula identified as C_30_H_26_O_13_ through accurate mass measurement analysis. The tandem mass spectra revealed distinctive product ions at *m/z* 285 [M-H-308Da]^−^, indicative of kaempferol. The corresponding fragmentation patterns are illustrated in Scheme 39, in the supporting information. Drawing upon the present MS/MS data and corroborating with the literature^41^, compound 54 was provisionally recognized as tiliroside.

Compounds 30 and 31 were detected as an [M-H]^−^ ion at *m/z* 595.129 with the same molecular formula C_26_H_28_O_16_. These compounds, identified as isomers, exhibited a common characteristic product ion at *m/z* 300 [M-H-295Da]^−∙^, signifying the presence of the quercetin moiety through the loss of arabino glucoside (-C_11_H_19_O_9_). Based on the current MS/MS data and previously documented literature^42^, these entities were tentatively categorized as isomers of quercetin-3-*O*-arabinohexoside (Scheme 25, in the supporting information).

Compounds 33 (rt 14.067 min) and 35 (14.354 min) were shown identical precursor ions [M-H]^−^ at *m/z* 463.087 with the same molecular formula C_21_H_20_O_12_. These two compounds were identified as isomers both displaying the characteristic product ion at *m/z* 300 [M-H-163Da]^−·^ due to the elimination of glucosyl moiety (–C_6_H_11_O_5_^·^). Based on the present MS/MS data and findings in the literature^43^, these two compounds are likely isomers of quercetin-*O*-hexoside, and their fragmentation pathways are depicted in Scheme 26 in the supporting information.

Compounds 37 (rt 14.723 min) and 38 (rt 15.100 min) were identified by the same [M-H]^−^ ions at *m/z* 433.077, sharing the identical molecular formula C_20_H_18_O_11_. Analysis of their tandem mass spectral data revealed analogous product ions at *m/z* 300 [M-H-133Da]^−^· and 271 [M-H-133-29Da]^−^, arising from the sequential loss of a sugar moiety, such as arabinosyl (-C_5_H_9_O_4_^∙^), followed by an HCO moiety, respectively. Accordingly, based on the current MS/MS data and insights from the literature^44^, these compounds are likely isomers of guaijaverin (Scheme 38, supporting information).

Compounds 39 (rt 15.639 min), 41 (15.938 min), 43 (16.194 min), and 44 (16.255 min) were observed as [M-H]^−^ ions at *m/z* 447.092, with the common molecular formula C_21_H_20_O_11_. The tandem mass spectra of compounds 39 and 44 exhibited analogous characteristic product ions at *m/z* 284 [M-H-163Da]^−^, *m/z* 255 [M-H-163-29Da]^−^, and 227 [M-H-163-29-28Da]^−^, attributed to the loss of the hexosyl moiety (-C_6_H_11_O_5_^·^), CO+H˙, and CO, respectively. On the contrary, compounds 41 and 43 generated indistinguishable product ions at *m/z* 300 [M-H-147Da]^·−^, 271 [M-H-147-29Da]^−^, and 243 [M-H-147-29-28Da]^−^, resulting from the loss of rhamnosyl, CO + H˙, and CO, respectively. Through a comprehensive analysis integrating current MS/MS data and corroborating literature sources^45,46^, compounds 39 and 44 were conclusively identified as isomers of kaempferol-*O*-hexoside (Ex: Astragalin), while 41 and 43 were unequivocally characterized as isomers of quercetin-*O*-rhamnoside (Ex: quercitrin). The fragmentation patterns of these compounds are presented in Scheme 44 and 33, in the supporting information.

Compounds 42 (rt 16.120 min) and 47 (16.581 min) were both detected with an identical precursor [M-H]^−^ ion at *m/z* 579.135, with the same molecular formula C_26_H_28_O_15_. These compounds discerned as isomers, exhibited similar characteristic product ions at 300 [M-H-279Da]^−^ and 301 [M-H-278Da]^−^, arising from the loss of neutral sugar moieties, specifically rhamnosyl-arabinopyranoside in radical and neutral form, respectively. Based on acquired MS/MS data and referencing pertinent literature^47–49^, both compounds were unequivocally identified as isomers of quercetin-*O*-rhamnosyl-arabinopyranoside. The corresponding fragmentation pattern is illustrated in scheme 29 (supporting information).

Compounds 46 and 49 exhibited a common precursor [M-H]^−^ ion at *m/z* 417.0820, sharing the molecular formula C_20_H_18_O_10_, yet eluted at distinct retention times of 16.463 min and 16.728 min, respectively. These compounds were identified as isomers, displaying an identical precursor ion at *m/z* 284 [M-H-132Da]^·−^ through the loss of the arabinosyl moiety. Upon further fragmentation, they yielded product ions at 255 [M-H-132-29Da]^−^ and 227 [M-H-132-29-28Da]^−^ by the neutral loss of CO + H^∙^ and CO molecules, respectively. According to the reported literature^50,51^ and the acquired MS/MS data, these two compounds were identified as isomers of kaempferol-*O*-arabinoside, and the fragmentation patterns of these compounds are presented in scheme 30 [supporting information].

Compound 50 (rt 17.977 min) was observed as an [M-H]^−^ ion at *m/z* 563.1399, and its molecular formula was elucidated through precise mass measurements as C_26_H_28_O_14_. The tandem mass spectra unveiled a fragment ion at *m/z* 431 [M-H-132Da]^−^, attributed to the detachment of the rhamnosyl moiety (-C_5_H_8_O_4_). Subsequent fragmentation led to ions at 284 [M-H-147Da]^−^ and 255 [M-H-132–147-29Da]^−^, arising from the loss of the hexosyl moiety (-C_6_H_11_O_4_˙) and CO + H˙, respectively. Based on the current MS/MS data (Scheme 31 [supporting information]) and corroborating with published ligature^52,53^, compound 50 was tentatively identified as kaempferol-*O*-rhamnoside-*O*-xyloside.

Compounds 51 (rt 18.287 min) and 53 (18.644 min) were both identified with an identical [M-H]^−^ ion at *m/z* 431.098, sharing the molecular formula C_21_H_20_O_10_. These compounds, recognized as isomers, exhibited similar MS/MS spectra, with product ions observed at *m/z* 284 [M-H-147Da]˙^−^, 255 [M-H-147-29Da]^−^, and 227 [M-H-147-29Da]^−^ resulting from the loss of rhamnosyl (-C_6_H_11_O_4_), CO + H˙, and CO, respectively. Utilizing the current MS/MS data (Scheme 32 [supporting information]) and referencing reported literature^54^, these compounds were conclusively identified as isomers of kaempferol-*O*-rhamnoside (Afzelin-I).

Compound 52 (rt 18.586 min) displayed a parent ion peak [M-H]^−^ at *m/z* 301.0350, corresponding to the molecular formula (C_15_H_10_O_7_). In the MS/MS spectra, characteristic fragment ions were observed at *m/z* 151 [M-H-150Da]^−^ due to the neutral loss of the glycosyl moiety (-C_8_H_6_O_3_) through RDA fission. Subsequently, it further fragmented into *m/z* 107 [M-H-150–44Da]^−^ by the loss of a CO_2_ group. The tentative identification of the compound as quercetin was established through a comparative analysis with reported literature^55^ and the current MS/MS data (Scheme 37) [supporting information].

Compound 55 (22.489 min) was identified with an [M-H]^−^ ion at *m/z* 723.1711, and its molecular formula was determined to be C_39_H_32_O_14_ through accurate mass measurement analysis. The tandem mass spectra of this compound revealed a distinctive fragment ion at *m/z* 285 [M-H-439Da]^−^, corresponding to the kaempferol moiety. Additionally, fragment ions at *m/z* 559 [M-H-164Da]^−^, 163 [M-H-560Da]^−^, and 119 [M-H-560-44Da]^−^ were observed, and the fragmentation patterns are depicted in Scheme 34 [supporting information]. Based on current MS/MS data and an online library match, this compound was tentatively identified as 2″,3″-di-*O*-*p*-coumaroylafzelin/platanoside^56^.

*Phenylpropanoid glycosides*. Compound 14 (rt: 8.65 min) was tentatively identified as citrusin F by comparing current HRMS data with previously reported literature and online databases (PubChem and HMDB). It was detected as an [M-H]^−^ ion at m/z 519.1712, and the molecular formula was determined to be C_22_H_32_O_14_ using ESI-HRMS analysis. Compound 15 (rt: 9.324 min) was detected as an [M-H]^−^ ion at *m/z* 441.1758, and the molecular formula C_21_H_30_O_10_ was determined based on accurate mass measurement. This compound was tentatively identified as lusitanicoside by comparing its MS data with previously reported literature^57^ and online databases [PubChem and HMDB].

*Miscellaneous compounds.* Compound 7 (rt 4.464 min) was tentatively identified as leonuriside A, based on information from previously reported literature^58^. It was observed as an [M-H]^−^ ion at *m/z* 331.1028 with the molecular formula C_14_H_20_O_9_. The MS/MS spectra unveiled a distinctive fragment ion at *m/z* 168 [M-H-163Da]^·-^, indicative of the loss of the hexose (-C_6_H_11_O_5_) moiety in the radical form, and further fragmented into *m/z* 153 [M-H-163–15Da]^·-^ by the loss of methyl radical (-CH_3_˙), as illustrated in Scheme 7 [supporting information]. Compound 13, with a retention time of 8.571 min, was identified as an [M-H]^−^ ion with a *m/z* value of 401.1456. The accurate mass measurement led to the determination of its molecular formula as C_18_H_26_O_10_. Further analysis through MS/MS spectra revealed a characteristic product ion at *m/z* 269 [M-H-132Da]^−^, indicative of the neutral loss of one of the sugar moieties (-C_5_H_9_O_4_). Based on comprehensive high-resolution MS and MS/MS fragmentation patterns [Scheme 13 (supporting information)], this compound was tentatively identified as benzyl *ꞵ*-primeveroside^59^.

Compound 27 (rt 11.88 min) manifested as an [M-H]^−^ ion at *m/z* 523.2177, with the ascribed molecular formula C_26_H_36_O_11_. The tandem mass spectral analysis revealed a distinctive fragment ion at *m/z* 361 [M-H-162Da]^−^, attributable to the neutral loss of a hexosyl moiety (-C_6_H_10_O_5_). Additionally, it underwent further fragmentation into *m/z* 346 through the loss of a methyl radical moiety [-CH_3_^·^]. Based on the current MS/MS data and corroborating literature^60^, this compound was tentatively identified as mascaroside [Scheme 27 (supporting information)].

Compound 56, eluting with a retention time of 24.87 min, was detected as [M-H]^−^ ion at *m/z* 297.2433, and its molecular formula was determined to be C_18_H_34_O_3_ using precise mass measurements from HRMS analysis. The tandem mass spectra revealed a distinctive fragment at *m/z* 183 [M-H-C_7_H_14_O]^−^, indicative of γ-hydrogen transfer through McLafferty rearrangement. Consistent with the available MS/MS data [Scheme 40 (supporting information)] and reported literature^61^, this compound has been tentatively identified as ricinoleic acid.

## Conclusion

This study offers the first comprehensive analysis of the chemical composition of traditionally used *Neolitsea pallens* leaves. A total of 56 compounds, including four organic acids, five phenolic acids and derivatives, six tannins, two phenylpropanoid glycosides, four lignan glycosides, thirty-one flavonoids, and four miscellaneous compounds, were identified. Utilizing UPLC-QTOF-MS/MS enabled detailed compound characterization by matching their fragmentation patterns with databases and literature. Among these, phenolics, represented by tannins, phenylpropanoids, lignans, and flavonoids, constituted the major class of compounds, which hold promise for various fields such as pharmaceuticals, nutraceuticals, and cosmetics. Moving forward, future studies should focus on exploring the specific biological activities and potential applications of these compounds. By probing deeper into the pharmacological and therapeutic properties of *N. pallens* leaves constituents, new opportunities for drug development, dietary supplements, and skincare products can be uncovered. Overall, our findings pave the way for interdisciplinary investigations aimed at harnessing the full potential of *N. pallens* leaves in diverse applications.

### Supplementary Information

Supplementary Information.

## Data Availability

The data that support the findings of this study are available from the corresponding author upon reasonable request.
