# Supplementary material for: Phytochemical exploration of Neolitsea pallens leaves using UPLC-Q-TOF-MS/MS approach
Source: Sci Rep. 2024 Apr 2;14:7770. doi: 10.1038/s41598-024-58282-6 (PMC10987493; doi:10.1038/s41598-024-58282-6)
Supplement: Supplementary file 1 — Supplementary Information. [file 41598_2024_58282_MOESM1_ESM.doc]

**Supporting Information**

**Phytochemical Exploration of *Neolitsea pallens* Leaves Using UPLC-Q-TOF-MS/MS Approach**

**Nisha Thakur^1^, K. Murali^1*^, Khushaboo Bhadoriya^1^, Y. C. Tripathi^1,*^ and V. K. Varshney^1*^**

***^1^Chemistry and Bio-prospecting Division, Forest Research Institute, Dehradun, India***

RESULTS AND DISCUSSIONS

| **Compound no.** | **Retention time (Min.)** | **[M-H]^-^ m/z** | **Experimental mass** | **Theoretical mass** | **Mass error (ppm)** | **Molecular formula** | **Fragment ions** | **Identified compounds** | **Class of compound** |
| --- | --- | --- | --- | --- | --- | --- | --- | --- | --- |
| 1. | 0.755 | 191.0197 | 192.0270 | 192.0197 | 0 | C_6_H_8_O_7_ | 173  155*  111  85 | Citric acid | Organic acid |

| **A** |
| --- |
| **B** |
| **C** |
| **D**  **Figure S1: MS spectrum (A), MS/MS spectrum at 3 different collision energy 10eV (B), 20eV (C) and 40 eV (D) of Citric acid.** |
| **Scheme 1:** (Fragmentation Pathway of Citric acid). |

| 2. | 0.833 | 133.0141 | 134.0213 | 134.0215 | -1.49 | C_4_H_6_O_5_ | 115*  71 | Malic acid | Organic acid |
| --- | --- | --- | --- | --- | --- | --- | --- | --- | --- |

| **A** |
| --- |
| **B** |
| **C** |
| **D**  **Figure S2: MS spectrum (A), MS/MS spectrum at 3 different collision energy 10eV (B), 20eV (C) and 40 eV (D) of Malic acid.** |
| **Scheme 2:** (Fragmentation Pathway of Malic acid). |

| 3. | 1.240 | 203.0192 | 204.0265 | 204.0270 | -2.4506 | C_7_H_8_O_7_ | 184  140  97* | Daucic acid | Organic acid |
| --- | --- | --- | --- | --- | --- | --- | --- | --- | --- |

| **A** |
| --- |
| **B** |
| **C** |
| **D**  **Figure S3: MS spectrum (A), MS/MS spectrum at 3 different collision energy 10eV (B), 20eV (C) and 40 eV (D) of Daucic acid.** |
| **Scheme 3:** (Fragmentation pathway of Daucic acid). |

| 4. | 1.371 | 117.0191 | 118.0264 | 118.0266 | -1.6945 | C_4_H_6_O_4_ | 99*  73 | Succinic acid | Organic acid |
| --- | --- | --- | --- | --- | --- | --- | --- | --- | --- |

| **A** |
| --- |
| **B** |
| **C** |
| **D**  **Figure S4: MS spectrum (A), MS/MS spectrum at 3 different collision energy 10eV (B), 20eV (C) and 40 eV (D) of Succinic acid.** |
| **Scheme 4:** (Fragmentation pathway of Succinic acid). |

| 5. | 2.450 | 191.0558 | 192.0630 | 192.0631 | -1.5619 | C_7_H_12_O_6_ | 173  129*  111 | Quinic acid | Phenolic acids |
| --- | --- | --- | --- | --- | --- | --- | --- | --- | --- |

| **A** |
| --- |
| **B** |
| **C** |
| **D**  **Figure S5: MS spectrum (A), MS/MS spectrum at 3 different collision energy 10eV (B), 20eV (C) and 40 eV (D) of Quinic acid.** |
| **Scheme 5:** (Fragmentation pathway of Quinic acid). |

| 6. | 2.633 | 331.0664 | 332.0737 | 332.0743 | -1.8068 | [C](https://pubchem.ncbi.nlm.nih.gov/#query=C13H16O10)_13_H_16_O_10_ | 168*  169  125  124 | 3-Glucogallic acid | Tannin |
| --- | --- | --- | --- | --- | --- | --- | --- | --- | --- |

| **A** |
| --- |
| **B** |
| **C** |
| **D**  **Figure S6: MS spectrum (A), MS/MS spectrum at 3 different collision energy 10eV (B), 20eV (C) and 40 eV (D) of 3-Glucogallic acid.** |
| **Scheme 6:** (Fragmentation pathway of 3-Glucogallic acid). |

| 7. | 4.464 | 331.1028 | 332.1099 | 332.1107 | -2.4088 | C_14_H_20_O_9_ | 168  153* | Leonuriside A | Phenolic glycosides |
| --- | --- | --- | --- | --- | --- | --- | --- | --- | --- |

| **A** |
| --- |
| **B** |
| **C** |
| **D**  **Figure S7: MS spectrum (A), MS/MS spectrum at 3 different collision energy 10eV (B), 20eV (C) and 40 eV (D) of Leonuriside A.** |
| **Scheme 7:** (Fragmentation pathway of Leonuriside A). |

| 8. | 6.893 | 457.1344 | 458.1417 | 458.1424 | -1.5279 | C_20_H_26_O_12_ | 163*  119 | cis-p-coumaric acid 4-[apiosyl-(1->2)-hexoside] | Phenolic acids derivative |
| --- | --- | --- | --- | --- | --- | --- | --- | --- | --- |

| **A** |
| --- |
| **B** |
| **C** |
| **D**  **Figure S8: MS spectrum (A), MS/MS spectrum at 3 different collision energy 10eV (B), 20eV (C) and 40 eV (D) of cis-*p*-Coumaric acid 4-[apiosyl-(1->2)-glucoside].** |
| **Scheme 8:** (Fragmentation pathway of cis-*p*-Coumaric acid 4-[apiosyl-(1->2)-glucoside). |

| 9. | 7.158 | 353.0872 | 354.0945 | 354.0950 | -1.4120 | C_16_H_18_O_9_ | 191*  163 | Chlorogenic acid | Phenolic acid |
| --- | --- | --- | --- | --- | --- | --- | --- | --- | --- |

| **A** |
| --- |
| **B** |
| **C** |
| **D**  **Figure S9: MS spectrum (A), MS/MS spectrum at 3 different collision energy 10eV (B), 20eV (C) and 40 eV (D) of Chlorogenic acid.** |
| **Scheme 9:** (Fragmentation pathway of Chlorogenic acid). |

| 10. | 7.220 | 577.1345 | 578.1418 | 578.1424 | -1.0378 | C_30_H_26_O_12_ | 425  407  289*  125 | Procyanidin B5 or Procyanidin B8 | Condensed Tannin |
| --- | --- | --- | --- | --- | --- | --- | --- | --- | --- |

| **A** |
| --- |
| **B** |
| **C** |
| **D**  **Figure S10: MS spectrum (A), MS/MS spectrum at 3 different collision energy 10eV (B), 20eV (C) and 40 eV (D) of Procyanidin B5 or Procyanidin B8.** |
| **Scheme 10:** (Fragmentation pathway of Procyanidin B5 or Procyanidin B8). |

| 11. | 7.364 | 863.1828 | 864.1893 | 864.1901 | -0.9257 | C_45_H_36_O_18_ | 711  411  289* | Procyanidin type 1A1B (Isomer-1) | Condensed Tannin |
| --- | --- | --- | --- | --- | --- | --- | --- | --- | --- |

| **A** |
| --- |
| **B** |
| **C** |
| **D**    **Figure S11: MS spectrum (A), MS/MS spectrum at 3 different collision energy 10eV (B), 20eV (C) and 40 eV (D) of Procyanidin type 1A1B (Isomer-1).** |
| **Scheme 11:** Fragmentation pathway of Procyanidin type 1A1B (Isomer-1). |

| 12. | 7.966 | 385.1133 | 386.1207 | 386.1212 | -1.2949 | C_17_H_22_O_10_ | 223*  179 | 1-*O*-Sinapoyl glucose (Isomer-1) | Phenolic acid derivative |
| --- | --- | --- | --- | --- | --- | --- | --- | --- | --- |

| **A** |
| --- |
| **B** |
| **C** |
| **D**  **Figure S12: MS spectrum (A), MS/MS spectrum at 3 different collision energy 10eV (B), 20eV (C) and 40 eV (D) of 1-O-Sinapoyl glucose (Isomer-1).** |
| **Scheme 12:** Fragmentation pathway of 1-O-Sinapoyl glucose (Isomer-1). |

| 13. | 8.571 | 401.1456 | 402.1522 | 402.1525 | -0.7459 | C_18_H_26_O_10_ | 269* | Benzyl *β*-primeveroside | Benzyl glycoside derivative |
| --- | --- | --- | --- | --- | --- | --- | --- | --- | --- |

| **A** |
| --- |
| **B** |
| **C** |
| **D**  **Figure S13: MS spectrum (A), MS/MS spectrum at 3 different collision energy 10eV (B), 20eV (C) and 40 eV (D) of Benzyl *β*-primeveroside.** |
| **Scheme 13:** (Fragmentation pathway of Benzyl *β*-primeveroside). |

| 14. | 8.625 | 519.1712 | 520.1784 | 520.1792 | -1.5379 | C_22_H_32_O_14_ | 342 | Citrusin F | Phenylpropanoid glycoside |
| --- | --- | --- | --- | --- | --- | --- | --- | --- | --- |

| **A** |
| --- |
| **B** |
| **C** |
| **D**  **Figure S14: MS spectrum (A), MS/MS spectrum at 3 different collision energy 10eV (B), 20eV (C) and 40 eV (D) of Citrusin F.** |
| **Scheme 14:** (Fragmentation pathway of Citrusin F). |

| 15. | 9.324 | 441.1758 | 442.1831 | 442.1838 | -1.5830 | C_21_H_30_O_10_ | 133 | Lusitanicoside | Phenylpropanoid glycoside |
| --- | --- | --- | --- | --- | --- | --- | --- | --- | --- |

| **A** |
| --- |
| **B** |
| **C** |
| **D**  **Figure S15: MS spectrum (A), MS/MS spectrum at 3 different collision energy 10eV (B), 20eV (C) and 40 eV (D) of Lusitanicoside.** |
| **Scheme 15: (**Fragmentation Pathway of Lusitanicoside). |

| 16. | 9.426 | 385.1134 | 386.1208 | 386.1212 | -1.0359 | C_17_H_22_O_10_ | 325  223  179 | 1-*O*-Sinapoyl glucose (Isomer -II) | Phenolic acid derivatives |
| --- | --- | --- | --- | --- | --- | --- | --- | --- | --- |

| **A** |
| --- |
| **B** |
| **C** |
| **D**  **Figure S16: MS spectrum (A), MS/MS spectrum at 3 different collision energy 10eV (B), 20eV (C) and 40 eV (D) of 1-*O*-Sinapoyl glucose (Isomer -II).** |
| **Scheme 16:** Fragmentation pathway of isomer of 1-*O*-Sinapoyl glucose (Isomer -II). |

| 17. | 9.648 | 521.2019 | 522.2091 | 522.2101 | -1.9149 | [C](https://pubchem.ncbi.nlm.nih.gov/#query=C26H34O11)_26_H_34_O_11_ | 359*  344  313 | Isolariciresinol-*O*-glucoside (Isomer-I) | Lignan glycoside |
| --- | --- | --- | --- | --- | --- | --- | --- | --- | --- |

| **A** |
| --- |
| **B** |
| **C** |
| **D**  **Figure S17: MS spectrum (A), MS/MS spectrum at 3 different collision energy 10eV (B), 20eV (C) and 40 eV (D) of Isolariciresinol-*O*-glucoside (Isomer-I).** |
| **Scheme 17.** Fragmentation pathway of Isolariciresinol-*O*-glucoside (Isomer-I). |

| 18. | 9.751 | 521.2020 | 522.2094 | 522.2101 | -1.3404 | C_26_H_34_O_11_ | 359  329* | Isolariciresinol-*O*-glucoside (Isomer-II) | Lignan glycoside | 9.751 |
| --- | --- | --- | --- | --- | --- | --- | --- | --- | --- | --- |

| **A** |
| --- |
| **B** |
| **C** |
| **D**  **Figure S18: MS spectrum (A), MS/MS spectrum at 3 different collision energy 10eV (B), 20eV (C) and 40 eV (D) of**  **Isolariciresinol-*O*-glucoside (Isomer-II).** |
| **Scheme 18.** Fragmentation pathway of Isolariciresinol-*O*-glucoside (Isomer-II). |

| 29. | 12.789 | 521.2020 | 522.2094 | 522.2101 | -1.3404 | C_26_H_36_O_11_ | 359*  329  341 | Isolariciresinol-*O*-glucoside (Isomer-III) | Stilbene glycosides |
| --- | --- | --- | --- | --- | --- | --- | --- | --- | --- |

| **A** |
| --- |
| **B** |
| **C** |
| **D**  **Figure S29: MS spectrum (A), MS/MS spectrum at 3 different collision energy 10eV (B), 20eV (C) and 40 eV (D) of Isolariciresinol*O*-glucoside (Isomer-III).** |
| **Scheme 19.** Fragmentation pathway of Isolariciresinol*O*-glucoside (Isomer-III) |

| 19. | 10.133 | 609.1476 | 610.1548 | 610.1533 | 2.4584 | C_27_H_30_O_16_ | 300*  271  255  151 | Quercetin-*O*- rhamnoside -hexoside (I)/ Rutin-I | Flavonol |
| --- | --- | --- | --- | --- | --- | --- | --- | --- | --- |

| **A** |
| --- |
| **B** |
| **C** |
| **D**  **Figure S19: MS spectrum (A), MS/MS spectrum at 3 different collision energy 10eV (B), 20eV (C) and 40 eV (D) of Quercetin-*O*- rhamnoside -hexoside (I)/ Rutin-I.** |

| 20. | 10.199 | 609.1454 | 610.1527 | 610.1533 | -0.9833 | [C](https://pubchem.ncbi.nlm.nih.gov/#query=C27H30O16)_27_H_30_O_16_ | 300*  255  271  178  151 | Quercetin-*O*- rhamnoside -hexoside (II)/ Rutin-II | Flavonol |
| --- | --- | --- | --- | --- | --- | --- | --- | --- | --- |

| **A** |
| --- |
| **B** |
| **C** |
| **D**  **Figure S20: MS spectrum (A), MS/MS spectrum at 3 different collision energy 10eV (B), 20eV (C) and 40 eV (D) of Quercetin-*O*- rhamnoside -hexoside (II)/ Rutin-II.** |

| 32. | 13.820 | 609.1451 | 610.1527 | 610.1533 | -0.9833 | C_27_H_30_O_16_ | 300*  301  463 | Quercetin-*O*- rhamnoside -hexoside (III)/ Rutin-III | Flavonol |
| --- | --- | --- | --- | --- | --- | --- | --- | --- | --- |

| **A** |
| --- |
| **B** |
| **C** |
| **D**  **Figure S32: MS spectrum (A), MS/MS spectrum at 3 different collision energy 10eV (B), 20eV (C) and 40 eV (D) of Quercetin-*O*- rhamnoside -hexoside (III)/ Rutin-III.** |

| 36. | 14.479 | 609.1456 | 610.1528 | 610.1533 | -0.8194 | C_27_H_30_O_16_ | 300*  271  151 | Quercetin-*O*- rhamnoside -hexoside (IV)/ Rutin-IV | Flavonol |
| --- | --- | --- | --- | --- | --- | --- | --- | --- | --- |

| **A** |
| --- |
| **B** |
| **C** |
| **D**  **Figure S36: MS spectrum (A), MS/MS spectrum at 3 different collision energy 10eV (B), 20eV (C) and 40 eV (D) of Quercetin-*O*- rhamnoside -hexoside (IV)/ Rutin-IV.** |
| **Scheme 36:** (Fragmentation pathway of Quercetin-3-*O*-arabinohexoside (Isomer-I), Quercetin-*O*- rhamnoside -hexoside (II)/ Rutin-II, Quercetin-*O*- rhamnoside -hexoside (III)/ Rutin-III and Quercetin-*O*- rhamnoside hexoside (IV)/ Rutin-IV). |

| 21. | 10.898 | 581.2231 | 582.2305 | 582.2312 | -1.2022 | C_28_H_38_O_13_ | 419  404 | Lyoniresinol 9-glucoside | Lignan glycosides |
| --- | --- | --- | --- | --- | --- | --- | --- | --- | --- |

| **A** |
| --- |
| **B** |
| **C** |
| **D**  **Figure S21: MS spectrum (A), MS/MS spectrum at 3 different collision energy 10eV (B), 20eV (C) and 40 eV (D) of Lyoniresinol 9-glucoside).** |
| **Scheme 20: (**Fragmentation pathway of Lyoniresinol 9-glucoside). |

| 22. | 10.91 | 331.0456 | 332.0528 | 332.0532 | -1.2046 | C_16_H_12_O_8_ | 300  271  178  151 | Laricitrin | Flavonol |
| --- | --- | --- | --- | --- | --- | --- | --- | --- | --- |

| **A** |
| --- |
| **B** |
| **C** |
| **D**  **Figure S22: MS spectrum (A), MS/MS spectrum at 3 different collision energy 10eV (B), 20eV (C) and 40 eV (D) of Laricitrin.** |
| **Scheme 21: (**Fragmentation pathway of Laricitrin). |

| 23. | 11.258 | 575.1193 | 576.1262 | 576.1265 | -0.5207 | C_30_H_24_O_12_ | 449  423  285*  289  125 | Procyanidin A2 | Proanthocyanidin |
| --- | --- | --- | --- | --- | --- | --- | --- | --- | --- |

| **A** |
| --- |
| **B** |
| **C** |
| **D**  **Figure S23: MS spectrum (A), MS/MS spectrum at 3 different collision energy 10eV (B), 20eV (C) and 40 eV (D) of Proanthocyanidin A2.** |
| **Scheme 22:** (Fragmentation pathway of Proanthocyanidin A2). |

| 24. | 11.398 | 863.1822 | 864.1894 | 864.1901 | -0.8100 | C_45_H_36_O_18_ | 711  575  451  287  125 | Procyanidin type 1A1B (Isomer-II) | Condensed Tannin |
| --- | --- | --- | --- | --- | --- | --- | --- | --- | --- |

| **A** |
| --- |
| **B** |
| **C** |
| **D**  **Figure S24: MS spectrum (A), MS/MS spectrum at 3 different collision energy 10eV (B), 20eV (C) and 40 eV (D) of Procyanidin type 1A1B (Isomer-II).** |
| **Scheme 24:** Fragmentation pathway of Procyanidin type 1A1B (Isomer-II). |

| 25. | 11.723 | 593.1511 | 594.1580 | 594.1584 | -0.6732 | C_27_H_30_O_15_ | 447  327  285* | Kaemferol-O-rhamnosyl-O-hexoside, isomer-I/ Astragalin 7-rhamnoside | Flavone |
| --- | --- | --- | --- | --- | --- | --- | --- | --- | --- |

| **A** |
| --- |
| **B** |
| **C** |
| **D**  **Figure S25: MS spectrum (A), MS/MS spectrum at 3 different collision energy 10eV (B), 20eV (C) and 40 eV (D) of Kaemferol-O-rhamnosyl-O-hexoside, isomer-I/ Astragalin 7-rhamnoside.** |

| 26. | 11.865 | 593.1505 | 594.1578 | 594.1584 | -1.0098 | C_27_H_30_ O_15_ | 285  255  161 | Kaemferol-O-rhamnosyl-O-hexoside, isomer-II/ Astragalin 7-rhamnoside | Flavonol |
| --- | --- | --- | --- | --- | --- | --- | --- | --- | --- |

| **A** |
| --- |
| **B** |
| **C** |
| **D**  **Figure S26: MS spectrum (A), MS/MS spectrum at 3 different collision energy 10eV (B), 20eV (C) and 40 eV (D) of** **Kaemferol-O-rhamnosyl-O-hexoside, isomer-II/ Astragalin 7-rhamnoside.** |
| **Scheme 26:** Fragmentation pathway of Kaemferol-O-rhamnosyl-O-hexoside/ Astragalin 7-rhamnoside (Isomer-I & II). |

| 28. | 11.950 | 577.1344 | 578.1417 | 578.1424 | -1.2107 | C_30_H_26_O_12_ | 451  425  407  289  125 | Procyanidin B8 or Procyanidin B5 | Condensed Tannin |
| --- | --- | --- | --- | --- | --- | --- | --- | --- | --- |

| **A** |
| --- |
| **B** |
| **C** |
| **D**  **Figure S28: MS spectrum (A), MS/MS spectrum at 3 different collision energy 10eV (B), 20eV (C) and 40 eV (D) of Procyanidin B8 or Procyanidin B5.** |
| **Scheme 28** (Fragmentation pathway of Procyanidin B8 or Procyanidin B5). |

| 30. | 12.967 | 595.1296 | 596.1371 | 596.1377 | -1.0064 | [C](https://pubchem.ncbi.nlm.nih.gov/#query=C26H28O16)_26_H_28_O_16_ | 300* | Quercetin-3-*O*-arabinohexoside (Isomer-I) | Flavonol |
| --- | --- | --- | --- | --- | --- | --- | --- | --- | --- |

| **A** |
| --- |
| **B** |
| **C** |
| **D**  **Figure S30: MS spectrum (A), MS/MS spectrum at 3 different collision energy 10eV (B), 20eV (C) and 40 eV (D) of Quercetin-3-*O*-arabinohexoside (Isomer-I).** |

| 31. | 13.161 | 595.1297 | 596.1369 | 596.1377 | -1.3419 | C_26_H_28_O_16_ | 300* | Quercetin-3-*O*-arabinohexoside (Isomer-II) | Flavonol |
| --- | --- | --- | --- | --- | --- | --- | --- | --- | --- |

| **A** |
| --- |
| **B** |
| **C** |
| **D**  **Figure S31: MS spectrum (A), MS/MS spectrum at 3 different collision energy 10eV (B), 20eV (C) and 40 eV (D) of Quercetin-3-*O*-arabinohexoside (Isomer-II).** |
| **Scheme 25:** Fragmentation pathway of Quercetin-3-*O*-arabinohexoside (Isomer-I & Isomer-II). |

| 33. | 14.067 | 463.0879 | 464.0951 | 464.0954 | -0.6464 | C_21_H_20_O_12_ | 300*  271  151 | Quercetin-*O*-hexoside, isomer-I | Flavonol |
| --- | --- | --- | --- | --- | --- | --- | --- | --- | --- |

| **A** |
| --- |
| **B** |
| **C** |
| **D**  **Figure S33: MS spectrum (A), MS/MS spectrum at 3 different collision energy 10eV (B), 20eV (C) and 40 eV (D) of Quercetin-*O*-hexoside, isomer-I.** |

| 35. | 14.354 | 463.0875 | 464.0947 | 464.0954 | -1.5083 | [C](https://pubchem.ncbi.nlm.nih.gov/#query=C21H20O12)_21_H_20_O_12_ | 300*  271  179  151 | Quercetin-*O*-hexoside , isomer-II | Flavonol |
| --- | --- | --- | --- | --- | --- | --- | --- | --- | --- |

| **A** |
| --- |
| **B** |
| **C** |
| **D**  **Figure S35: MS spectrum (A), MS/MS spectrum at 3 different collision energy 10eV (B), 20eV (C) and 40 eV (D) of** **Quercetin-*O*-hexoside, isomer-II.** |
| **Scheme 35:** (Fragmentation pathway followed by Quercetin-*O*-hexoside, isomer-I & isomer-II). |

| 34. | 14.188 | 593.1503 | 594.1576 | 594.1584 | -1.3464 | C_27_H_30_O_15_ | 447  284*  255  163 | Kaempferol-*O*-rhamnosyl-hexoside, Isomer-I | Flavonol |
| --- | --- | --- | --- | --- | --- | --- | --- | --- | --- |

| **A** |
| --- |
| **B** |
| **C** |
| **D**    **Figure S34: MS spectrum (A), MS/MS spectrum at 3 different collision energy 10eV (B), 20eV (C) and 40 eV (D) of Kaemferol-*O*-rhamnosyl-hexoside, Isomer-I.** |

| 40. | 15.717 | 593.1501 | 594.1576 | 594.1584 | -1.3464 | C_27_H_30_O_15_ | 447  285* | Kaempferol-*O*-rhamnosyl-hexoside, Isomer-II | Flavonol |
| --- | --- | --- | --- | --- | --- | --- | --- | --- | --- |

| **A** |
| --- |
| **B** |
| **C** |
| **D**  **Figure S40: MS spectrum (A), MS/MS spectrum at 3 different collision energy 10eV (B), 20eV (C) and 40 eV (D) of Kaemferol-*O*-rhamnosyl-hexoside, Isomer-II.** |

| 45. | 16.416 | 593.1502 | 594.1575 | 594.1584 | -1.5147 | C_27_H_30_O_15_ | 447  285*  255 | Kaempferol-*O*-rhamnosyl-hexoside, Isomer-III | Flavonol |
| --- | --- | --- | --- | --- | --- | --- | --- | --- | --- |

| **A** |
| --- |
| **B** |
| **C** |
| **D**  **Figure S45: MS spectrum (A), MS/MS spectrum at 3 different collision energy 10eV (B), 20eV (C) and 40 eV (D) of Kaemferol-*O*-rhamnosyl-hexoside, Isomer-III.** |

| 48. | 16.606 | 593.1504 | 594.1577 | 594.1584 | -1.1781 | C_27_H_30_O_15_ | 285*  255  151 | Kaempferol-*O*-rhamnosyl-hexoside, Isomer-IV | Flavonol |
| --- | --- | --- | --- | --- | --- | --- | --- | --- | --- |

| **A** |
| --- |
| **B** |
| **C** |
| **D**  **Figure S45: MS spectrum (A), MS/MS spectrum at 3 different collision energy 10eV (B), 20eV (C) and 40 eV (D) of Kaemferol-*O*-rhamnosyl-hexoside, Isomer-IV.** |
| **Scheme 23:** (Fragmentation pathway of Kaempferol-*O*-rhamnosyl-hexoside, Isomer-I, II, III & IV). |

| 37. | 14.723 | 433.0771 | 434.0843 | 434.0849 | -1.3822 | C_20_H_18_O_11_ | 300*  271 | Guaijaverin (Isomer-1) | Flavonol |
| --- | --- | --- | --- | --- | --- | --- | --- | --- | --- |

| **A** |
| --- |
| **B** |
| **C** |
| **D**  **Figure S37: MS spectrum (A), MS/MS spectrum at 3 different collision energy 10eV (B), 20eV (C) and 40 eV (D) of Guaijaverin (Isomer-I).** |

| 38. | 15.100 | 433.0770 | 434.0843 | 434.0849 | -1.3822 | C_20_H_18_O_11_ | 300*  271  178  151 | Guaijaverin (Isomer-2) | Flavonol |
| --- | --- | --- | --- | --- | --- | --- | --- | --- | --- |

| **A** |
| --- |
| **B** |
| **C** |
| **D**  **Figure S38: MS spectrum (A), MS/MS spectrum at 3 different collision energy 10eV (B), 20eV (C) and 40 eV (D) of** **Guaijaverin (Isomer 2).** |
| **Scheme 38:** (Fragmentation pathway of Guaijaverin Isomer 1 and 2). |

| 39. | 15.639 | 447.0928 | 448.1001 | 448.1005 | -0.8926 | C_21_H_20_O_11_ | 447  284*  255  227 | Kaempferol -*O*-hexoside (Isomer-I) | Flavonol |
| --- | --- | --- | --- | --- | --- | --- | --- | --- | --- |

| **A** |
| --- |
| **B** |
| **C** |
| **D**  **Figure S39: MS spectrum (A), MS/MS spectrum at 3 different collision energy 10eV (B), 20eV (C) and 40 eV (D) of** **Kaempferol-*O*-hexoside (Isomer-I)** |

| 44. | 16.255 | 447.0934 | 448.1006 | 448.1005 | 0.2232 | C_21_H_20_O_11_ | 285  284*  255  227 | Kaempferol -*O*-hexoside (Isomer-II**)** | Flavonol |
| --- | --- | --- | --- | --- | --- | --- | --- | --- | --- |

| **A** |
| --- |
| **B** |
| **C** |
| **D**  **Figure S44: MS spectrum (A), MS/MS spectrum at 3 different collision energy 10eV (B), 20eV (C) and 40 eV (D) of Kaempferol -*O*-hexoside (Isomer-II).** |
| **Scheme 44:** Fragmentation pathway of Kaempferol-*O*-hexoside (Isomer-I & II**).** |

| 41. | 15.938 | 447.0926 | 448.0998 | 448.1005 | -1.5621 | C_21_H_20_O_11_ | 301*  300  271  243 | Quercetin-*O*-rhamnoside (Isomer-I) | Flavonol |
| --- | --- | --- | --- | --- | --- | --- | --- | --- | --- |

| **A** |
| --- |
| **B** |
| **C** |
| **D**  **Figure S41: MS spectrum (A), MS/MS spectrum at 3 different collision energy 10eV (B), 20eV (C) and 40 eV (D) of Quercetin-*O*-rhamnoside (Isomer-I).** |

| 43. | 16.194 | 447.0930 | 448.1003 | 448.1005 | -0.4463 | C_21_H_20_O_11_ | 301*  271  243 | Quercetin -*O*-rhamnoside (Isomer-II) | Flavonol |
| --- | --- | --- | --- | --- | --- | --- | --- | --- | --- |

| **A** |
| --- |
| **B** |
| **C** |
| **D**  **Figure S43: MS spectrum (A), MS/MS spectrum at 3 different collision energy 10eV (B), 20eV (C) and 40 eV (D) of Quercetin-*O*-rhamnoside (Isomer-II).** |
| **Scheme 33:** (Fragmentation pathway of Quercetin-*O*-rhamnoside (Isomer-II & II). |

| 42. | 16.120 | 579.1353 | 580.1425 | 580.1428 | -0.5171 | C_26_H_28_O_15_ | 300*  271 | Quercetin-*O* -rhamnosyl-arabinopyranoside (Isomer-I) | Flavonol |
| --- | --- | --- | --- | --- | --- | --- | --- | --- | --- |

| **A** |
| --- |
| **B** |
| **C** |
| **D**  **Figure S42: MS spectrum (A), MS/MS spectrum at 3 different collision energy 10eV (B), 20eV (C) and 40 eV (D) of Quercetin-*O*-rhamnosyl-arabinopyranoside (Isomer-I).** |

| 47. | 16.581 | 579.1348 | 580.1421 | 580.1428 | -1.2066 | C_26_H_28_O_15_ | 447  300 | Quercetin-*O*-rhamnosyl-arabinopyranoside (Isomer-II) | Flavonol |
| --- | --- | --- | --- | --- | --- | --- | --- | --- | --- |

| **A** |
| --- |
| **B** |
| **C** |
| **D**  **Figure S47: MS spectrum (A), MS/MS spectrum at 3 different collision energy 10eV (B), 20eV (C) and 40 eV (D) of Quercetin-*O*-rhamnosyl-arabinopyranoside (Isomer-II).** |
| **Scheme 29:** Fragmentation pathway of Quercetin-*O*-rhamnosyl-arabinopyranoside (Isomer-I & II). |

| 46. | 16.463 | 417.0820 | 418.0895 | 418.0899 | -0.9567 | C_20_H_18_O_10_ | 284*  255  227 | Kaempferol-*O*-arabinoside (Isomer-I) | Flavonol |
| --- | --- | --- | --- | --- | --- | --- | --- | --- | --- |

| **A** |
| --- |
| **B** |
| **C** |
| **D**  **Figure S46: MS spectrum (A), MS/MS spectrum at 3 different collision energy 10eV (B), 20eV (C) and 40 eV (D) of Kaempferol-*O*-arabinoside (Isomer-I).** |

| 49. | 16.728 | 417.0820 | 418.0892 | 418.0899 | -1.6742 | C_20_H_18_O_10_ | 284*  255  227 | Kaempferol-*O*-arabinoside (Isomer-II) | Flavonol |
| --- | --- | --- | --- | --- | --- | --- | --- | --- | --- |

| **A** |
| --- |
| **B** |
| **C** |
| **D**  **Figure S49: MS spectrum (A), MS/MS spectrum at 3 different collision energy 10eV (B), 20eV (C) and 40 eV (D) of Kaempferol-*O*-arabinoside (Isomer-II).** |
| **Scheme 30:** Fragmentation pathway of Kaempferol-*O*-arabinoside (Isomer-I & Isomer-II). |

| 50. | 17.977 | 563.1399 | 564.1472 | 564.1479 | -1.2408 | C_26_H_28_O_14_ | 284*  255 | Kaempferol-*O*-rhamnoside-*O*-xyloside | Flavonol |
| --- | --- | --- | --- | --- | --- | --- | --- | --- | --- |

| **A** |
| --- |
| **B** |
| **C** |
| **D**  **Figure S50: MS spectrum (A), MS/MS spectrum at 3 different collision energy 10eV (B), 20eV (C) and 40 eV (D) of Kaempferol-*O*-rhamnoside-*O*-xyloside.** |
| **Scheme 31:** (Fragmentation pathway of Kaempferol-*O*-rhamnoside-*O*-xyloside). |

| 51. | 18.287 | 431.0978 | 432.1050 | 432.1056 | -1.3855 | C_21_H_20_O_10_ | 285*  255  227 | Kaempferol -*O*-rhamnoside, Isomer-I/Afzelin-I | Flavonol |
| --- | --- | --- | --- | --- | --- | --- | --- | --- | --- |

| **A** |
| --- |
| **B** |
| **C** |
| **D**  **Figure S51: MS spectrum (A), MS/MS spectrum at 3 different collision energy 10eV (B), 20eV (C) and 40 eV (D) of Kaempferol -*O*-rhamnoside, Isomer-I/Afzelin-I.** |

| 53. | 18.644 | 431.0982 | 432.1054 | 432.1056 | -0.4628 | C_21_H_20_O_10_ | 285*  255  227 | Kaempferol -*O*-rhamnoside, Isomer-II/Afzelin-I | Flavonol |
| --- | --- | --- | --- | --- | --- | --- | --- | --- | --- |

| **A** |
| --- |
| **B** |
| **C** |
| **D**  **Figure S53: MS spectrum (A), MS/MS spectrum at 3 different collision energy 10eV (B), 20eV (C) and 40 eV (D) of Kaempferol -*O*-rhamnoside, Isomer-II/Afzelin-I.** |
| **Scheme 32**: Fragmentation pathway of Kaempferol -*O*-rhamnoside/Afzelin-I (Isomer-I & II). |

| 52. | 18.586 | 301.0350 | 302.0422 | 302.0422 | 0 | C_15_H_10_O_7_ | 178  151* | Quercetin | Flavonol |
| --- | --- | --- | --- | --- | --- | --- | --- | --- | --- |

| **A** |
| --- |
| **B** |
| **C** |
| **D**  **Figure S52: MS spectrum (A), MS/MS spectrum at 3 different collision energy 10eV (B), 20eV (C) and 40 eV (D) of Quercetin.** |
| **Scheme 37:** (Fragmentation pathway of Quercetin). |

| 54. | 20.121 | 593.1290 | 594.1361 | 594.1373 | -2.0197 | C_30_H_26_O_13_ | 285*  284*  165  121 | Tiliroside | Flavonol |
| --- | --- | --- | --- | --- | --- | --- | --- | --- | --- |

| **A** |
| --- |
| **B** |
| **C** |
| **D**  **Figure S54: MS spectrum (A), MS/MS spectrum at 3 different collision energy 10eV (B), 20eV (C) and 40 eV (D) of Tiliroside.** |
| **Scheme 39:** (Fragmentation pathway of Tiliroside). |

| 55. | 22.489 | 723.1711 | 724.1783 | 724.1792 | -1.2427 | C_39_H_32_O_14_ | 285*  559 | 2'',3''-Di-*O*-*p*-coumaroylafzelin/ Platanoside | Flavonol |
| --- | --- | --- | --- | --- | --- | --- | --- | --- | --- |

| **A** |
| --- |
| **B** |
| **C** |
| **D**  **Figure S55: MS spectrum (A), MS/MS spectrum at 3 different collision energy 10eV (B), 20eV (C) and 40 eV (D) of 2'',3''-Di-*O*-*p*-coumaroylafzelin/ Platanoside.** |
| **Scheme 34:** (Fragmentation pathway of 2'',3''-Di-*O*-*p*-coumaroylafzelin/ Platanoside). |

| 27. | 11.881 | 523.2177 | 524.2252 | 524.2257 | -0.9537 | C_26_H_36_O_11_ | 361*  315  161 | Mascaroside | Naphthofuran |
| --- | --- | --- | --- | --- | --- | --- | --- | --- | --- |

| **A** |
| --- |
| **B** |
| **C** |
| **D**  **Figure S27: MS spectrum (A), MS/MS spectrum at 3 different collision energy 10eV (B), 20eV (C) and 40 eV (D) of Mascaroside.** |
| **Scheme 27:** (Fragmentation pathway of Mascaroside). |

| 56. | 24.87 | 297.2433 | 298.2505 | 298.2507 | -0.6705 | [C](https://pubchem.ncbi.nlm.nih.gov/#query=C18H34O3)_18_H_34_O_3_ | 183* | Ricinoleic acid | Fatty acid |
| --- | --- | --- | --- | --- | --- | --- | --- | --- | --- |

| **A** |
| --- |
| **B** |
| **C** |
| **D**  **Figure S56: MS spectrum (A), MS/MS spectrum at 3 different collision energy 10eV (B), 20eV (C) and 40 eV (D) of Ricinoleic acid.** |
| **Scheme 40:** (Fragmentation pattern of Ricinoleic acid). |
